# A scoping review of prehabilitation interventions for arthroplasty patients

**DOI:** 10.4102/sajp.v79i1.1939

**Published:** 2023-11-14

**Authors:** Prithi Pillay-Jayaraman, Verusia Chetty, Stacy Maddocks

**Affiliations:** 1 Chris Hani Baragwanath Academic Hospital, Faculty of Health, Gauteng Department of Health, Johannesburg, South Africa; 2 Department of Physiotherapy, Faculty of Health Sciences, University of KwaZulu-Natal, Durban, South Africa; 3 Department of Physiotherapy, Faculty of Health Sciences, University of the Witwatersrand, Johannesburg, South Africa

**Keywords:** prehabilitation, arthroplasty, exercise, rehabilitation, lower limb, program design

## Abstract

**Background:**

Osteoarthritis (OA) is a long-term condition that causes significant impairment, and because of the increasing prevalence of OA, the demand for arthroplasty will continue to rise. However, the demand will not be matched by availability, because of prioritisation of trauma-related surgeries. Implementing prehabilitation could assist physiotherapists in having an impact on improving access by reducing the length of stay.

**Objectives:**

The aim of our scoping review was to explore, map and identify trends and gaps to better inform the content of a prehabilitation programme.

**Method:**

In our scoping review, studies between 1995 and 2020 were identified and included based on inclusion and exclusion criteria and study methodology described by Arksey and O’Malley. The results were collated and summarised as a narrative synthesis.

**Results:**

A total of 200 articles were identified and exported from four databases of which 48 articles were included in the final analysis. Regarding the efficacy of prehabilitation interventions, 21 studies reported significant results supporting prehabilitation, whereas 11 studies reported non-significant results.

**Conclusions:**

Prehabilitation could be a valuable adjunct in reducing length of hospital stay and improving functional outcomes in adults undergoing total joint replacement.

**Clinical implications:**

The scoping review described the information available on prehabilitation in lower limb arthroplasty patients and could potentially inform the design of a prehabilitation programme suitable for use in the South African public health context.

## Background

Osteoarthritis (OA) is a long-term condition that causes significant impairment of physical function in individuals. Globally, prevalent cases of OA increased by 113.25%, from 247.51 million in 1990 to 527.81 million in 2019 (Long et al. 2022). The highest numbers of prevalent cases in 2019 were observed in China (132.81 million), followed by India (62.36 million) and the United States (51.87 million), with corresponding percentage changes from 1990 to 2019 of 156.58%, 165.75% and 79.63%, respectively (Long et al. 2022). Osteoarthritis presents a burden on individuals, health systems and social care systems, with further indirect cost implications (Woolf & Pfleger 2003), and in most cases, OA has clear predisposing risk factors, such as genetics, trauma, ageing or obesity (He et al. 2020). Studies from the United States report that OA affects approximately 10% of non-institutionalised adults, resulting in substantial clinical, humanistic and economic burdens (Zhao et al. 2019). A study based in Canada reports that from 2010 to 2031, the prevalence of OA was estimated to increase from 13.8% to 18.6%, with an almost 2.6-fold cost increase in the management of these patients (Sharif et al. 2015). In terms of the cost components that will constitute the total direct cost of OA in 2031 are hospitalisation cost, outpatient services, alternative care and out-of-pocket cost categories, drugs, rehabilitation and side effects of drugs (Sharif et al. 2015).

The 2017 Global Burden of Disease report ranked hip and knee OA as the 11th-highest contributor to global disability and the 23rd-highest cause of disability-adjusted life years. A study done to investigate the burden of OA in the Middle East and North Africa (MENA) reported that there was an increase in the burden of OA from 1990 to 2019 in the MENA region with a reported increase in the incidence of OA by 9.4% from 1990 to 2019 (Shamekh et al. 2022). The study emphasises the importance of early preventative approaches to control any future health, economic and quality of life crises imposed by OA in this region (Shamekh et al. 2022).

The effect of arthritis in higher income countries is reduced workplace productivity; however, in low- and middle-income countries (LMICs), arthritis imposes a potential additional burden by creating a vicious cycle that subsequently worsens poverty (Brennan-Olsen et al. 2017). And yet it is reported that LMICs have 90% of the global burden of disease but only 12% of global health spending (Brennan-Olsen et al. 2017). The situation is similar in the South African public health sector, which spends 4.5% of its gross domestic product on health and serves 86% of the population (Motsoaledi 2018), which worsens the impact of arthritis. In South Africa, of all the types of arthritis, OA is the most prevalent with a prevalence of 55.1% (95% confidence interval [CI]: 40.74–73.54) among adults aged 65 years and older in an urban setting; with knee OA prevalence of 33.1% (95% CI: 27.70–38.50) among adults aged 35 years and older in a rural setting (Usenbo et al. 2015). However, there is a paucity of data later than 2015 in South Africa.

Because of ageing populations, the obesity epidemic and an increasing prevalence of OA, the demand for total hip arthroplasty (THA) and total knee arthroplasty (TKA) as a treatment of choice for OA will continue to increase (Kurtz et al. 2009). A recent study conducted in the United States forecasts an increase in the demand for THA procedures to be 176% by 2040 and 659% by 2060 and the increase for TKA to be 139% by 2040 and 469% by 2060 (Shichman et al. 2023). Despite the lack of South African-based studies forecasting the future need for arthroplasty, from the global trends, one may anticipate that the need will similarly increase in the South African public and private sectors. The popularity and demand for replacement arthroplasties is due to its success rates and excellent outcomes, achieved with THA surgeries, specifically, being referred to as the ‘operation of the century’ (Learmonth, Young & Rorabeck 2007).

However, in LMICs like South Africa, because of political prioritisation, the peremptory nature of emergency care, trauma and infective pathology, trauma caseloads dominate the workload of the public sector, which serves the majority of the population, and elective procedures like replacement arthroplasty take a back seat and are not prioritised, resulting in the long waiting lists for the procedure (Abera Abaerei, Ncayiyana & Levin 2017). In a South African-based study of surgical procedures, 54.2% were emergency surgeries with a risk of admission to critical care and 23.7% were leading to the conclusion that most patients in South Africa’s public sector hospitals undergo urgent and emergency surgery (Biccard & Madiba 2015).

The quiet suffering of the functionally impaired patient with arthritis is largely not significant for immediate intervention, based on the assumption that leaving patients with degenerative joint diseases like arthritis to deteriorate and living with pain is less important that other prioritised healthcare needs like emergency work (Dunn 2012). Because of the discrepancies between demand and available resources in the public health sphere in South Africa, patients wait years for arthroplasty procedures. According to the waiting list registry of the arthroplasty unit at the public sector Chris Hani Baragwanath Academic Hospital, a patient assessed as needing an arthroplasty in 2020 could potentially only receive the surgery in 2023 (Dunn 2012; Kavalieratos, Nortje & Dunn 2017). The estimated expenditure of uncomplicated hip arthroplasty in a tertiary public hospital in South Africa is ZAR74 185.25 with a minimum and maximum of ZAR60 414.04 and ZAR110 598.62, respectively (Sekeitto & Aden 2021), which implies that arthroplasty surgery makes a significant dent in the health expenditure budget. There is a paucity of further academic data regarding cost of knee replacement, complicated surgeries and costing of all cost-driven procedures within the orthopaedic field (Sekeitto & Aden 2021).

Patients waiting for elective surgery in the United Kingdom report a significant impact on their health status and quality of life, with 71.2% reporting a further deterioration in their condition while waiting and 6.3% evaluating their health status as ‘worse than death’ (Morris et al. 2020). This necessitates consideration of all measures to improve access as an urgent imperative, especially in the face of the recent coronavirus-19 (COVID-19) pandemic, which resulted in waiting lists for elective surgeries becoming even longer (Morris et al. 2020).

One of the ways in which a physiotherapist can assist in improving access to arthroplasty surgery is by implementing measures to decrease the postoperative length of hospital stay, which also has an impact on reducing costs. A broad overview of systematic reviews, and meta-analysis, allowed for the conclusion that some of the studies support the practice of prehabilitation programmes because of their effect on pain, range of motion, physical function, quality of life, length of hospital stay and postoperative outcomes in individuals awaiting joint replacement (Gill & McBurney 2013; Moyer et al. 2017; Vasta et al. 2020; Wallis & Taylor 2011). However, other studies conclude that prehabilitation programmes not effective in improving outcomes (Almeida, Khoja & Zelle 2020; Barbay 2009; Chen et al. 2018; Peer et al. 2017) suggest the need for a thorough exploration of individual studies and a descriptive narrative of the literature to draw a more informed conclusion on the topic. Preoperative optimisation with a multidisciplinary team has been suggested as a need in South Africa with the specific aim to ensure patients are physiologically and mentally fit for surgery (Plenge et al. 2020). The study recommended that the preoperative period be a ‘window of opportunity’ in a South African setting with low literacy levels and limited ability to seek information. A pragmatic approach to patient education would improve patient empowerment and participation in postoperative rehabilitation (Plenge et al. 2020). The long waiting period gives credence to this rationale.

The preliminary review of the literature allowed for the identification of gaps in prehabilitation programmes, including limitations regarding the types of exercises prescribed, with specific reference to the exclusion of foot exercises, balance and proprioceptive exercises, despite anecdotal and clinical observations of the presence of foot deformities, like flat feet and hallux valgus (HV). In terms of the content of the education provided in a South African setting, the overview of the literature highlights a lack of pain neuroscience education (Saw et al. 2016). A detailed synthesis of the literature will inform other aspects of prehabilitation programmes, such as the minimum number of sessions needed for clinically significant results, the mode of delivery and the potential need for intensive face-to-face sessions weeks prior to the surgery. In the context of a resource-constrained country like South Africa, there is a need for delivering effective prehabilitation programmes as transport cost and time spent waiting in hospitals have an impact on patients accessing hospital services (Abera Abaerei et al. 2017). The principles enshrined in the Batho Pele concepts of healthcare delivery dictate that value for money be an important consideration for healthcare professionals when delivering services (James & Miza 2015). Our scoping review may also inform and strengthen the formulation of a standard package of care for arthroplasty patients in public hospitals in South Africa, prompting the need for all relevant literature to be exhaustively analysed, critiqued and described, prior to constructing the framework of the prehabilitation programme.

## Methodology

The protocol for our scoping review has been registered within the Open Science Framework platform (https://osf.io/9fdsh/), and our study protocol is being reported in accordance with the reporting guidance provided in the PRISMA extension for scoping reviews (PRISMA-ScR) (Online Appendix 1). The scoping review methodology, steps in defining the research question and selection of eligible studies have been described extensively in a previously published paper by the same authors (Pillay-Jayaraman, Maddocks & Chetty 2023).

In the search for literature, computer databases like Google Scholar, Pubmed, Ebsco and Cochrane Library were used, and any studies conducted between 1995 and 2020 were included in the search. The Boolean terms ‘AND’, ‘OR’ and ‘NOT’ were used to separate keywords. Search terms included were knee, hip, joint replacement; arthroplasty; physiotherapy; physical therapy; exercise; rehabilitation and prehabilitation. To synthesise the principles of exercise prescription from the literature, and to better inform the construction of a prehabilitation programme, those papers that elaborated and described aspects of exercises, prescribing aerobic, anaerobic, strengthening, flexibility, resistance, balance and functional activities, were included. The studies were also scrutinised for the principles of exercise prescription in terms of the frequency, intensity, time and type (FITT) and their correlation to efficacy and outcomes of treatment. Papers with content on education as a preparation, either as the sole model for prehabilitation or as an adjunct for joint arthroplasty optimisation, were also included.

**FIGURE 1:**
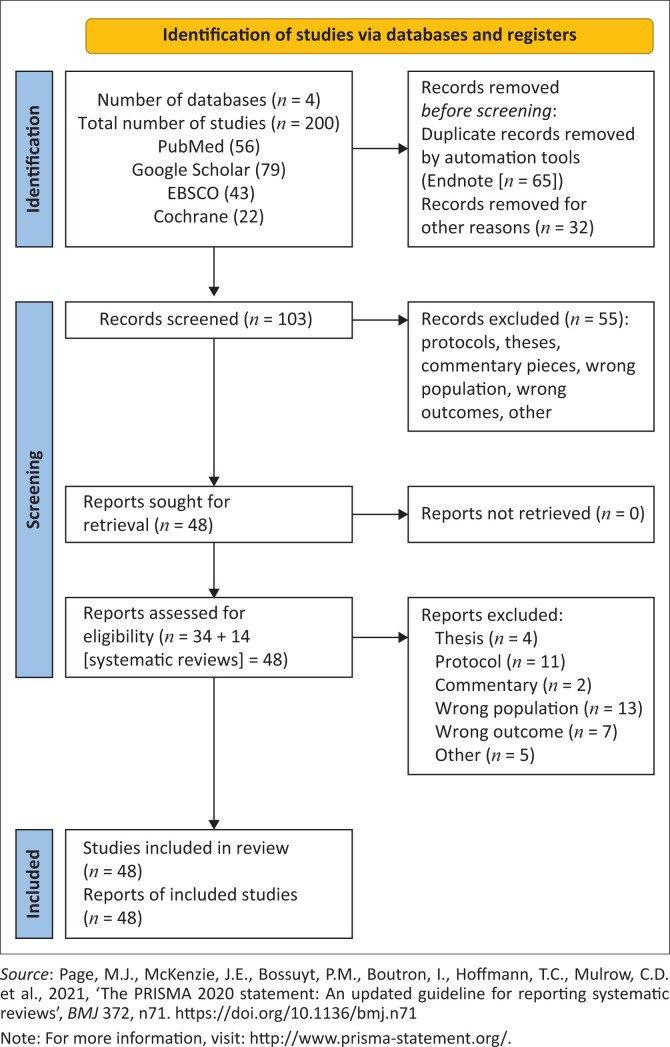
PRISMA flowchart of identification of studies via databases.

The above principles formed the basis for article selection and the databases that were searched and articles identified from each have been represented in Figure 1. Duplicate records were removed by automation tools such as EndNote (*n* = 65) before screening. The three reviewers who were familiar with the study protocol were involved in the scoping review process. Title, abstract and keywords screening of all eligible articles were conducted by the first author (P.P.) and second reviewer (H.E.). Excluded citations were reviewed and confirmed by a third reviewer (V.C.). The next step was to obtain full texts of all the selected articles by undertaking a thorough and exhaustive search of the web. In those instances where the full text could not be obtained from the web, a concerted effort was made to obtain these full-text articles by engaging with the university subject librarian and/or contacting the author(s) as necessary. Full-text screening to ascertain whether the selected articles met the inclusion criteria was conducted by all reviewers (P.P., H.E. and V.C.) independently. Major discrepancies and lack of agreement among reviewers (P.P. and H.E.) for inclusion in the scoping review were resolved through discussion.

Data from the studies were charted according to the information from randomised controlled trials and descriptive studies (Online Appendix 2) to gather as much detailed information as possible on the metrics used to describe prehabilitation in arthroplasty and to describe the interventions in detail to inform the development of a prehabilitation programme.

### Ethical considerations

This article does not contain any studies involving human participants performed by any of the authors.

## Results

A total of 200 articles were identified and exported to EndNote version 20.5. After the review of titles, and removal of duplicates, 103 articles were selected. Title and abstract screening resulted in 48 articles being included, while 55 articles were excluded, for reasons including that the articles were protocols, theses or commentary pieces, and had the wrong population or outcomes. Full-text articles were then retrieved and a final 48 articles were collated and charted to answer the research question at hand (Figure 1).

## Description of studies: Systematic reviews and meta-analyses

The literature search revealed that there were 8 systematic reviews and 6 meta-analyses, with 3 papers on total knee replacement and 11 on joint replacement. Six of the studies reported improvements in length of hospital stay (Almeida et al. 2020; Chen et al. 2018; Coudeyre et al. 2007; Moyer et al. 2017; Sharma, Ardebili & Abdulla 2019; Vasta et al. 2020), and two reported no improvement (Kwok, Paton & Haddad 2015; Wang et al. 2006). Of the systematic reviews that looked at functional outcomes, four studies reported no improvement in functional outcomes (Ackerman & Bennell 2004; Almeida et al. 2020; Kwok et al. 2015; Sharma et al. 2019); four reported improvements in outcomes (Chen et al. 2018; Moyer et al. 2017; Vasta et al. 2020; Wang et al. 2006); two reported improvements in pain (Gill & McBurney 2013; Wang et al. 2006); one reported improvements on postoperative recovery (Wang et al. 2006) and one study reported no improvement in isometric quadriceps muscle strength (Peer et al. 2017). The majority of the studies reported positive results in length of hospital stay, outcomes, pain, strength and functional recovery, following hip replacement. In terms of the results for knee replacement, one study found prehabilitation to be effective in reducing length of hospital stay (Chen et al. 2018) but two studies reported no effect of prehabilitation on quadriceps strength (Chen et al. 2018; Peer et al. 2017) and one study concluded that there was no added advantage of prehabilitation (Ma et al. 2018).

The results were not conclusive because of the randomised controlled trials not being well designed (Ma et al. 2018). There was no standardisation in the prehabilitation programmes, with variation in the prehabilitation protocols (Almeida et al. 2020; Chen et al. 2018; Gill & McBurney 2013). The studies had small sample size and a power analysis was not reported in most of the papers included in the study by Vasta et al. (2020). It was reported that the small sample size could lead to an unpredictable overestimation or underestimation of the results (Vasta et al. 2020). Recommendations stemming from the analysis of systematic reviews and the meta-analysis were that more research was needed to emphatically conclude on the effects of prehabilitation (Barbay 2009; Wallis & Taylor 2011). It is essential to develop and describe a comprehensive prehabilitation programme (Almeida et al. 2020) and assess the effectiveness of pre- and post-intervention when compiling and reporting on the effects of prehabilitation (Almeida et al. 2020). In addition, training intensity (Chen et al. 2018), appropriate dosage (Kwok et al. 2015) and compliance with therapy need to be reported to ensure desired outcomes of therapy are achieved (Wang et al. 2016). The studies also recommend that an intervention 6 weeks before the surgery may not yield sufficient, statistically significant, clinical gains (Kwok et al. 2015), and adherence may be affected by the need to travel frequently a few weeks prior to the surgery to attend face-to-face therapy. Hence, consideration of these factors may lead to a different picture and more conclusive answers. Another factor that is relevant in the context of a country like South Africa based on the limited research on the topic is that patients with knee arthritis had waited on average 461 (90–1513 [222.8]) days and hip replacements waited on average of 412 (104–1593 [242.3]) days (Kavalieratos et al. 2017). While this presents many challenges, it does on the other hand present the opportunity to have more time to optimise patients. In addition, patient’s experience of care, satisfaction and preparedness should also be considered in assessing effectiveness.

Table 1 is a description of study characteristics of randomised controlled trials and descriptive studies.

**TABLE 1:** Study characteristics of randomised controlled trials and descriptive studies (Online Appendix 2, Table 3).

Methodology types	Sample size	Study duration	Compliance	Mode of delivery	Results
RCT: 23 Prospective: 3 Retrospective: 3 Other: 5 (case studies, longitudinal cohorts, descriptive cross-sectional surveys observational cohorts)	Average: 110.6 Minimum: Case study (Ehrlich 2019): 1 Maximum: Survey design: 608 Mode: 20 and 35 patients	Average: 6.5 weeks Minimum: 6 weeks Maximum: 12 weeks Mode: 8 weeks	Average: 16.6 sessions Minimum: 6 sessions Maximum: 60 sessions Mode: 24 sessions	Home: 4 Face-to-Face: 20 Hybrid: 10	Statistically significant: 21 Not significant: 11

Note: Please see the article, Pillay-Jayaraman, P., Chetty, V. & Maddocks, S., 2023, ‘A scoping review of prehabilitation interventions for arthroplasty patients’, *South African Journal of Physiotherapy* 79(1), a1939. https://doi.org/10.4102/sajp.v79i1.1939, for more information on where to find Online Appendix 2.

RCT, randomised controlled trial.

## Description of exercise interventions

Twenty-nine studies used exercise-based interventions. However, two studies (Kosek et al. 2013; Topp et al. 2009) did not discuss the intervention in detail and two studies used hydrotherapy (Gill, McBurney & Schulz 2009; Sunghye et al. 2021) as a means of delivering exercises. The exercise programmes used could be categorised into isometric, range of motion, aerobic, strengthening, stretching and circuit exercises. Two studies targeted quadriceps for isometric contractions (Aytekin et al. 2019; Cavill et al. 2016) and seven studies incorporated range of motion exercises (Aytekin et al. 2019; Cavill et al. 2016; Desmeules, Hall & Woodhouse 2013; Doiron-Cadrin et al. 2020; Gill et al. 2009; Rooks et al. 2006; Sunghye et al. 2021). The most common joints targeted were the hip, knee and ankle, whereas the study by Rooks et al. (2006) incorporated single planar motion of the cervical spine, shoulders, elbows, wrists and hands (Rooks et al. 2006). Aerobic exercises were described in 13 studies and included a warm-up session (Brown et al. 2012; D’Lima et al. 1996; Doiron-Cadrin et al. 2020) of walking (Kim et al. 2020; McKay, Prapavessis & Doherty 2012; Swank et al. 2011) or used equipment like a stationary bike (Cavill et al. 2016; Desmeules et al. 2013; Gill et al. 2009; McKay et al. 2012; Moe & Dagfinrud 2014; Rooks et al. 2006; Villadsen et al. 2014) or arm ergometry (Calatayud et al. 2017). The main reason behind incorporating an aerobic session was to improve cardiovascular conditioning, maintain heart rate and improve general fitness (D’Lima et al. 1996; Kim et al. 2020). The majority of the studies did aerobic exercises for 10 min, increasing to a maximum time of 20 min (Kim et al. 2020).

Strengthening exercises were most frequently used, with 22 studies describing them as part of the prehabilitation programme regime. Muscles targeted for strengthening predominately included the abdominals and lower limb muscles like triceps surae, quadriceps, hamstrings, hip flexors, hip extensors, hip abductors and the calf muscles. In the study by Rooks et al. (2006), upper limb muscles like shoulder flexors, shoulder abductors and triceps brachii were also strengthened.

Stretching was used in 15 of the papers. In this context, the term ‘stretching’ was an umbrella term that included flexibility exercises and a way of cooling down. The stretching or cooling down was done for a minimum of 5 min (Calatayud et al. 2017) and a maximum of 15 min (Brown et al. 2012, 2014), with 2–3 repetitions held for 20 s – 30 s. Step training was another intervention utilised which included going up and down a single step, forward and then sideways to the left and right, and starting from a step height of 2 inches – 3 inches (5 cm – 7 cm), going up to 4 inches – 7 inches (Brown et al. 2014; Cavill et al. 2016; Swank et al. 2011). Other exercises included functional weight-bearing exercises which imitated functions of daily living and taught patients how to control hip-knee-foot alignment (Fernandes et al. 2017).

Circuit training was also used as an approach for preoperative optimisation in six studies (Clode, Perry & Wulff 2018; Huber et al. 2015; McKay et al. 2012; Moe & Dagfinrud 2014; Swank et al. 2011; Villadsen et al. 2014), using 4 (Huber et al. 2015) to 13 exercises (Clode et al. 2018). The main purpose was to improve core stability or postural function and orientation, functional alignment, lower extremity and muscle strength. Studies also used manual therapy, such as patellofemoral joint mobilisation (Cavill et al. 2016); balance (static and dynamic) (Denduluri, Huddleston & Amanatullah 2020), without and with a balance trainer (Calatayud et al. 2017); and proprioceptive exercises (Doiron-Cadrin et al. 2020). Relaxation and visualisation (Saw et al. 2016), ice pack (Beaupre et al. 2004) and transcutaneous electric nerve stimulation (Rahmatika, Novriansyah & Indriastuti 2020) and practicing with gait aids (Desmeules et al. 2013) were other techniques incorporated.

Compliance was noted by attendance and keeping exercise logs (Online Appendix 2, Table 3), and in the study by Saw et al. (2016), participants were required to set exercise and activity goals on a weekly basis and to record these in their workbooks (Saw et al. 2016). In the study by D’Lima et al. (1996), patients were taught to use a graphed exercise programme to meet a goal determined by the patient and physiotherapist, based on the patient’s capabilities. The graph was used to record the number of repetitions, which was increased by one repetition every third day until the goal was reached (D’Lima et al. 1996).

## Description of educational interventions

A total of 12 studies described educational interventions as a part of the prehabilitation programme. The overarching themes in the content of preoperative education were analysed. The main themes included preoperative preparation, hospital information, discharge information, postoperative management, patients’ expectations, precautions and contraindications, transfers, ambulation and assistive devices, the home environment, goal setting, surgery information and OA information.

There were four studies that included preoperative preparation as a part of the content and discussed with patients on how to prepare for surgery (Lewis et al. 2020; Rooks et al. 2006; Soeters et al. 2018; Tait, Dredge & Barnes 2015). In five studies, hospital information was included as a part of the content and informed patients on what to expect day one post-surgery, things to bring for the hospital stay and examples of exercises that would be prescribed after the surgery (Denduluri et al. 2020; Lewis et al. 2020; Rooks et al. 2006; Soeters et al. 2018; Tait et al. 2015). Five studies included discharge information as part of the content and elaborated on discharge destination and guidelines (Clode et al. 2018; Denduluri et al. 2020; Lewis et al. 2020; Tait et al. 2015). In six studies, postoperative management information such as information on postoperative pain management, details of postoperative rehabilitation, the postoperative range-of-motion routine and physiotherapist recommendations for recovery were discussed with patients (Denduluri et al. 2020; Huber et al. 2015; Soeters et al. 2018; Tait et al. 2015; Villadsen et al. 2014). The study by Soeters et al. (2018) was the only study in which patient expectations and goal setting were discussed (Soeters et al. 2018). In three studies, precautions and contraindications (Lewis et al. 2020; Soeters et al. 2018; Tait et al. 2015) were discussed and three studies discussed transfers (Aytekin et al. 2019; Soeters et al. 2018). In addition to education, the patient in one study also had an opportunity to practice transfers in and out of bed, a chair, a toilet seat or a car (Aytekin et al. 2019). Four studies discussed ambulation and assistive devices, where the options for ambulation with and without assistive devices, negotiation of stairs before surgery and crutch walking on ground level and on stairs were discussed (Aytekin et al. 2019; Lewis et al. 2020; Soeters et al. 2018). Six studies discussed home environment modifications with their patients, with specific reference to potential assistive equipment, safety recommendations for the home environment and modifying a home to improve accessibility (Aytekin et al. 2019; Denduluri et al. 2020; Lewis et al. 2020; Rooks et al. 2006; Soeters et al. 2018; Tait et al. 2015). Four studies discussed surgery information with their patients, including the risks of surgery (Aytekin et al. 2019; Denduluri et al. 2020; Tait et al. 2015; Villadsen et al. 2014), and four other studies discussed information regarding OA, the pathogenesis of advanced OA and disability (Aytekin et al. 2019; Clode et al. 2018; Gill et al. 2009; Saw et al. 2016).

## Discussion

In our review of 4376 participants in 34 randomised and descriptive studies, 21 studies reported that their results were significant, while 11 studies reported non-significant results. Because the majority of the studies reported a significant result, it allows for a conclusion that prehabilitation programmes are indeed a valuable adjunct to treatment, and this is in keeping with the review by Moyer et al. (2017), which analysed 35 individual studies (2956 subjects) and reported that a prehabilitation programme could improve physical function, quadricep strength, length of hospital stay and pain, after total joint replacement (Moyer et al. 2017). Similar findings were reported by Chen et al. (2018), and in this systematic review, 16 studies were analysed (966 subjects). The study reported that prehabilitation programmes had the potential to improve physical function, range of motion and length of hospital stay, after total knee replacement (Chen et al. 2018). There were two reviews that focused on length of hospital stay after total knee replacements and both studies reported that prehabilitation programmes had a positive effect, including reducing the length of hospital stay (Ma et al. 2018; Sharma et al. 2019).

The above results are, however, contrary to the finding of the review by Almeida et al. (2020), whose study concluded that it was unknown if prehabilitation programmes improved functional outcomes, as preoperative functional status had not been established. The review noted that if studies had not assessed the efficacy of prehabilitation programmes preoperatively, then it was not possible to assess the impact of the programme in improving strength and physical function postoperatively (Almeida et al. 2020). In our review, 33 studies were intervention-based studies and the majority (18) of those studies evaluated the efficacy of prehabilitation by assessing outcomes prior to the surgery. However, only 10 of the studies reported significant results at the end of the assessment period. The review by Almeida et al. (2020) also reported that none of the systematic reviews assessed compliance with the rehabilitation programme, unlike in the findings of our review, where compliance was recorded and reported in 21 studies (Almeida et al. 2020).

There was only one South African-based study (74 participants) in our review and the primary outcome focused on pain, measured by the brief pain inventory and an open-ended questionnaire assessing the patient’s experience (Saw et al. 2016). The results of the study showed that the intervention group had significant improvements compared to the control group, with moderate-to-large effect sizes (ES) on pain severity, and 53% of participants reported that the intervention decreased their pain. The study recommended that further research be conducted to explore long-term and postoperative outcomes (Saw et al. 2016). This was the only study that had a targeted intervention for the pain experienced with OA. It is understood and described in the literature that the pain experienced by patients with OA is a complex, subjective phenomenon, where each individual’s experience is unique and influenced by biological, psychological and social factors (Neogi 2013). Hence, pain neuroscience education and addressing the multifaceted nature of the pain experience should form an important part of any prehabilitation programme in order to have the desired positive outcome of the surgery.

The most utilised intervention to improve outcomes was the use of strengthening exercises. However, from anecdotal experience and the profile of the patients accessing the arthroplasty services in the public sector, the inclusion of balance and proprioception exercises is an important component of a comprehensive prehabilitation programme. Two studies incorporated balance exercises as part of their programme (Calatayud et al. 2017; Denduluri et al. 2020). The study by Denduluri et al. (2020) did not elaborate on the exact nature of the exercises; and in the study by Calatayud et al. (2017), participants performed four sets of 30 s double leg stance exercises and four sets of 15 s of single leg stance exercises on the same unstable device (Bosu^®^ Balance Trainer), starting with the non-affected leg. Proprioceptive exercises were included in two studies, but these studies did not describe the exercises in detail (Desmeules et al. 2013; Doiron-Cadrin et al. 2020). No studies specifically evaluated the efficacy of balance and proprioceptive exercises. However, a meta-analysis by Zhang and Xiao (2020) suggested that, in patients’ post-arthroplasty, balance and proprioceptive training after total joint arthroplasty improved self-reported functionality and balance, which were maintained mid-term (Zhang & Xiao 2020). In another synthesis, Domínguez-Navarro et al. (2018) concluded that, in clinical terms, balance training was a convenient adjunct to conventional physiotherapy care to improve balance and functionality after a knee replacement (Domínguez-Navarro et al. 2018). The inclusion of balance and proprioceptive exercises has also been studied in patients with OA, and the study by Duman et al. (2012) recommended that adding exercises specifically targeting the proprioceptive and balance dysfunction is beneficial for patients with advanced OA (Duman et al. 2012). In the systematic review by Smith, King and Hing (2012), it was concluded that there is some evidence to indicate the efficacy of proprioceptive exercises, compared to general strengthening exercises, in functional outcomes in patients with OA (Smith et al. 2012). The above indicates a definitive gap in the design of prehabilitation programmes, and that balance and proprioceptive exercises should be included as an integral component when designing a prehabilitation programme.

Balance and proprioception can also be impacted by foot pain and deformities and there has been a definitive link in the literature between these factors and OA. Prevalence and incidence studies have identified that severe foot pain is more common in obese women, aged 65–74 years, with hand or knee OA, who walk more slowly and perform the timed five sit to stand from a chair slower that the cohort average (Leveille et al. 1998). Wilder, Barrett and Farina (2005) found that, in both genders, there was a significant, positive relationship between grade 2+ foot OA and second distal interphalangeal joint, third proximal interphalangeal joint, first carpometacarpal joint and knee OA. This relationship remained significant after adjustment for age, body mass index (BMI) and occupational history (Wilder et al. 2005). Foot conditions like HV are prevalent in the community and are associated with age, the female sex and components of generalised OA, such as nodal OA, knee pain, big toe pain and self-reported OA. Hallux valgus poses a significant health problem and is associated with foot pain, poor balance, immobility and the risk of falling (Roddy, Zhang & Doherty 2008). In a study by Golightly et al. (2015), HV was associated with the female sex, African Americans, older individuals, pes planus and knee or hip OA. It was inversely associated with higher BMI in this population study (Golightly et al. 2015). In terms of foot problems and their correlation with balance, the study by Menz and Lord (2001) concluded that foot problems are common in older people and are associated with impaired balance and performance in functional tests. They also concluded that foot problems are a risk factor for falling and suggested that the cumulative effect of multiple foot problems is more important in increasing the risk of falling, than the presence or absence of individual foot conditions (Menz & Lord 2001). Similar profiles can be found in the public health population and anecdotal evidence exists to confirm a similar clinical picture in South Africa. Hence, the recommendation that there is a need to fill this gap in the literature by providing targeted interventions that address balance and proprioception is justified. In addition, foot deformities could be addressed by conservative management and the routine inclusion of podiatry for holistic management.

## Conclusion and recommendations

Prehabilitation could be a valuable adjunct in reducing the length of hospital stay and improving functional outcomes in adults undergoing total joint replacement. However, future studies must consider chronic pain neuroscience education, balance and proprioceptive training, as integral elements in the prehabilitation programme. In the South African public health context, which serves most of the population, the waiting lists for arthroplasty are extensive and there is certainly a huge need to optimise patients. Considering the economic challenges in communities, a hybrid approach of face-to-face therapy and telerehabilitation will serve to enhance compliance and patient activity, ensuring the successful implementation of the prehabilitation programme. Based on the scoping review, a possible ideal version of the programme is presented in Online Appendix 3. A potential limitation of the scoping review methodology is the fact that only English language papers were considered, notwithstanding further research is needed to determine the impact of language restriction on systematic reviews in particular fields of medicine.
